# Cardio-Cerebral Infarction, Free-Floating Thrombosis and Hyperperfusion in COVID-19

**DOI:** 10.3390/neurolint13020027

**Published:** 2021-06-11

**Authors:** Sitara Koneru, Dinesh V. Jillella, Raul G. Nogueira

**Affiliations:** 1 Department of Neurology, Emory University School of Medicine, Atlanta, GA 30322, USA; sitara.koneru@emory.edu (S.K.); raul.g.nogueira@emory.edu (R.G.N.); 2 Marcus Stroke and Neuroscience Center, Grady Memorial Hospital, Atlanta, GA 30303, USA

**Keywords:** stroke, COVID-19, thrombus, perfusion, neuroimaging

## Abstract

Cardio-cerebral infarction, which refers to an acute ischemic stroke (AIS) and acute myocardial infarction (AMI) that occur concurrently, is an uncommon phenomenon with a grave prognosis. Intraluminal carotid thrombus (ICT) is an infrequently encountered cause of ischemic stroke and can be associated with an underlying hypercoagulable state. One severe yet prevalent complication of infection with Coronavirus Disease 2019 (COVID-19) is thrombosis from multi-pathway inflammatory responses. Here, we present a unique case of cardio-cerebral infarction, with a free-floating intraluminal thrombus in the left internal carotid artery, in the setting of recent COVID-19 infection, and with the etiology of both events attributed to a COVID-19 hypercoagulable state. CT perfusion imaging also showed an interesting imaging finding of hyperperfusion, which is believed to be a form of dysfunctional cerebral autoregulation.

An early 50-year-old male with type 2 diabetes mellitus and hyperlipidemia presented with isolated transcortical sensory aphasia that correlated with a National Institute of Health Stroke Scale (NIHSS) of 3. CT angiography showed a free-floating intraluminal thrombus in the clinoid segment of the left internal carotid artery (Figure 1A). There was subtle hypervascularity of the anterior left middle cerebral artery (MCA) territory with an increase in regional cerebral blood flow (CBF) (Figure 1C) and cerebral blood volume (CBV), as well as decreased Tmax (Figure 1D) on perfusion imaging, consistent with hyperperfusion. Additionally, there was an area of decreased CBF and increased Tmax in the posterior left MCA territory, consistent with core infarction, which correlated to ischemic infarct area seen on MRI brain diffusion weighted imaging (Figure 1B). He did not receive acute reperfusion therapy due to presentation beyond the therapeutic window, as well as absence of favorable perfusion characteristics on initial imaging. Additionally, he was found to have an acute inferior-wall ST-elevation MI with initial troponin of 4.97 ng/mL that peaked at 10.79 ng/mL. Cardiac catheterization was deferred given the risk of hemorrhagic transformation of AIS with high doses of anticoagulation and dual anti-platelet therapy that would be needed. No contributive cardiac pathology was found on transthoracic echocardiography, cardiac CT with contrast, or on telemetry that was continued throughout admission. As an outpatient post hospital discharge, the patient had Holter monitoring that recorded about 117 hours of data and did not show any evidence of atrial fibrillation. Hypercoagulable work-up was completed both as an inpatient and also one month after hospital discharge and was unrevealing. The patient reported a recent COVID-19 exposure and clinical symptoms of chills in the days preceding this presentation, with pulmonary imaging showing peripheral ground-glass opacities in a pattern consistent with COVID-19 pneumonia. Swab-based PCR testing for COVID-19 was negative, but the IgG antibody test returned positive, suggesting recent infection. D-dimer was elevated at 1218 ng/mL on admission. He was initially treated with therapeutic anticoagulation with intravenous (IV) heparin; however this was discontinued 36 hours later due to petechial hemorrhage, as seen on repeat head imaging. He was subsequently treated with antiplatelet monotherapy. Magnetic resonance imaging (MRI) of the brain confirmed an AIS involving the left MCA territory (Figure 1B). He had an uncomplicated hospital course and was discharged in good condition with NIHSS of 2 for a mild aphasia. CT angiography of the neck was repeated one month after hospital discharge and showed resolution of ILT.

Simultaneous acute ischemic stroke (AIS) and acute myocardial infarction (AMI), also referred to as cardio-cerebral infarction, is an extremely rare entity with an estimated incidence of 0.009% [1]. Additionally, AIS, due to intraluminal carotid artery thrombus (ICT), is an infrequent condition and is often associated with ipsilateral carotid stenosis or underlying hypercoagulable state [2]. Infection with SARS-CoV-2, the causative organism for Coronavirus disease 2019 is thought to cause endothelial inflammation and a hypercoagulable state leading to both arterial and venous thrombotic events [3]. This case report discusses a unique case of simultaneous AIS from ICT and AMI in the setting of recent SARS-CoV-2 infection. Cardiac work-up in our patient did not reveal any abnormalities that could explain the concurrent infarcts and therefore, the etiology of both events was attributed to COVID-19 hypercoagulable state. Additionally, imaging studies showed a rare phenomenon of hyperperfusion that is likely unrelated to his recent COVID-19 infection but occurs when cerebral blood flow increases in the region of previous ischemia (hypoperfusion and/or infarcted brain tissue) and is thought to be a form of dysfunctional autoregulation [4]. While this typically occurs in the subacute phase, it can occur in the acute phase, such as with our patient, and in these instances is hypothesized to be due to revascularization after development of collateral flow. One limitation of this case report is that the COVID-19 infection was not confirmed by PCR testing. Given his recent infectious symptoms within the past week prior to hospital presentation, exposure to COVID-19 contacts two weeks prior, supportive chest imaging findings, positive IgG testing, it was thought that he had an acute COVID-19 infection, especially considering the probability of false negative results on PCR testing. Unfortunately, our center did not have the capability to test for COVID-19 IgM antibodies at that time to confirm the acuity of this infection. Our report highlights an atypical presentation of acute cardio-cerebral infarction with ICT and hyperperfusion in the setting of a recent SARS-CoV-2 infection.

## Figures and Tables

**Figure 1:**
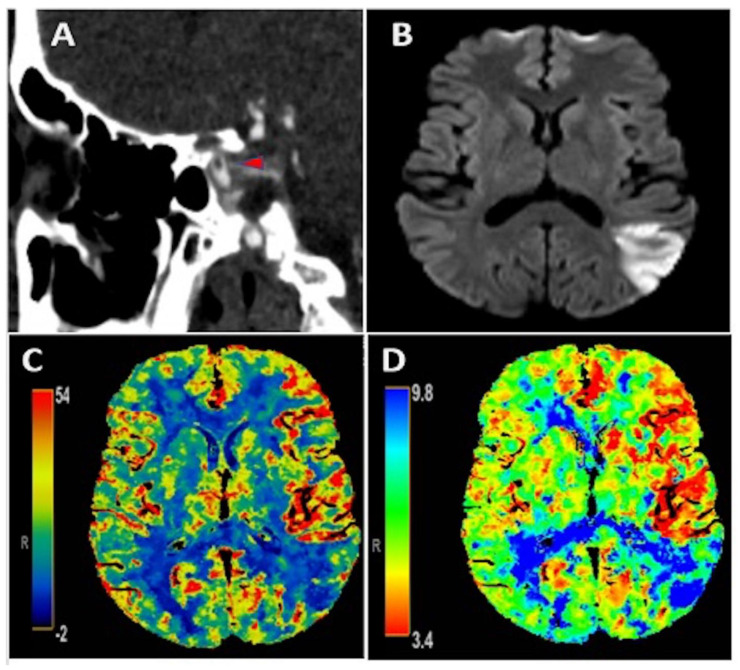
**Panel** (**A**): CT angiography showing an intraluminal free-floating thrombus (red arrowhead) in the clinoid portion of the left internal carotid artery; **Panel** (**B**): MRI brain diffusion weighted imaging illustrating an acute ischemic stroke in the posterior left middle cerebral artery (MCA) territory. **Panels** (**C**,**D**): CT perfusion depicting increased CBF and decreased Tmax, respectively, in the anterior left MCA territory consistent with hyperperfusion in this area as well as decreased CBF and increased Tmax in the posterior left MCA territory consistent with core infarction and corresponding with ischemic stroke seen on MRI brain in **Panel** (**B**).

## Data Availability

The data used to support the findings of this case report are available within the manuscript.
